# Physiological and Molecular Mechanisms Underlying the Differential Responses of Wheat Seedlings to Different Types of Salt Stress

**DOI:** 10.1002/fsn3.71454

**Published:** 2026-02-11

**Authors:** Duo Liu, Zheng Wang, Hongyao Lou, Ping Li, Kangfeng Cai, Wei Jiang, Zhaobo Chen

**Affiliations:** ^1^ Beijing Key Laboratory of Crop Molecular Design and Intelligent Breeding, Beijing Key Laboratory of Molecular Genetics in Hybrid Wheat Institute of Hybrid Wheat Beijing Academy of Agriculture and Forestry Science Xinxiang China; ^2^ Farmland Irrigation Research Institute Chinese Academy of Agricultural Sciences Xinxiang China; ^3^ Institute of Crop and Nuclear Technology Utilization Zhejiang Academy of Agricultural Sciences Hangzhou China; ^4^ Xianghu Laboratory Hangzhou China; ^5^ College of Agriculture Nanjing Agricultural University Nanjing China

**Keywords:** expression analysis, gene family, saline‐alkali stress, salt stress, *Triticum aestivum* L.

## Abstract

Soil salinization, driven by rapid climate change, poses a serious threat to wheat ( *Triticum aestivum* L.) production worldwide. The studies on the effect of sodium chloride stress on wheat have detailed reports, while the effects of Na_2_SO_4_, NaHCO_3_, and Na_2_CO_3_ stresses remain to be investigated. Here, we investigated the differential growth and physiological responses of wheat seedlings to equimolar concentrations of NaCl, Na_2_SO_4_, NaHCO_3_, and Na_2_CO_3_. Alkaline salts (NaHCO_3_ and Na_2_CO_3_) induced significantly more severe growth inhibition, chlorophyll degradation, and oxidative damage compared to neutral salts (NaCl and Na_2_SO_4_). This was evidenced by heightened lipid peroxidation, reactive oxygen species accumulation, and membrane injury, particularly under Na_2_CO_3_ stress. The antioxidant defenses were precisely tailored, which alkaline stress strongly activated ascorbate while neutral salts preferentially enhanced catalase activity. Osmotic adjustment was also stress‐specific, with alkaline conditions triggering extreme proline accumulation up to 7.5‐fold in roots. Ion homeostasis was profoundly disrupted under alkaline stress, marked by excessive Na^+^ uptake, severe K^+^ depletion, and significant reductions in nitrogen and phosphorus. Notably, gene expression analysis revealed stress‐specific regulation of key genes involved in ion transport (e.g., *SOS1*) and antioxidant defense. Our findings revealed distinct stress‐specific regulatory mechanisms in wheat, with alkaline causing more severe oxidative stress and membrane damage than salt. In addition, we examined the tissue expression and evolution of *SOD* genes, which showed the expansion and duplication of the *SOD* gene family in terrestrial plants. Our study unveils the divergent physiological pathways activated by different salts, providing novel insights into wheat stress adaptation and a theoretical basis for breeding salt‐tolerant cultivars.

## Graphical abstract

Chinese Spring (CS), Qing Mai 6 (QM), ascorbate peroxidase (APX), catalase (CAT), superoxide dismutase (SOD), Peroxidase(POD), Malondialdehyde, (MDA).

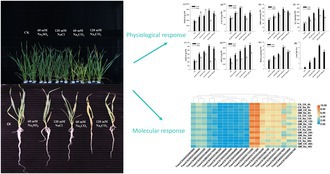

## 1 Introduction

Saline‐alkali soils contain a complex variety of soluble salts, such as sodium chloride (NaCl), sodium sulfate (Na_2_SO_4_), sodium bicarbonate (NaHCO_3_), and sodium carbonate (Na_2_CO_3_) (Cao et al. 2022; Li et al. 2024). The stress caused by NaCl and Na_2_SO_4_ is termed neutral salt stress, whereas that caused by NaHCO_3_ and Na_2_CO_3_ is called alkaline salt stress (Ganasula et al. 2025). Soil salinization is one of the most significant abiotic stresses worldwide (Daliakopoulos et al. 2016; Xiao and Zhou 2023), affecting approximately 80 million hectares of irrigated land (Mourad et al. 2023). With global climate change (Hu et al. 2023), seawater intrusion, and unreasonable artificial irrigation practices (Shaban et al. 2024), the area of salinized land continues to increase (Abdel Latef et al. 2019). Therefore, plants have developed diverse molecular and physiological mechanisms in response to salt and alkaline stress (Li et al. 2023; Liu et al. 2024).

Wheat ( *Triticum aestivum* L.) is an essential food crop and the primary source of carbohydrates for human consumption (Cui et al. 2023; Ji et al. 2024), making it a cornerstone of agricultural sustainability and dietary needs worldwide (Wang et al. 2024; He et al. 2025). Saline‐alkali stress can inhibit the germination and growth of wheat, compromising yield (Wang, Lv, et al. 2022). This reduction in yield has been attributed to the detrimental effects of high soil salinity on various plant physiological processes, including water uptake, nutrient absorption, and photosynthesis (Chen et al. 2021). Consequently, the decrease in yield and quality of wheat induces a substantial challenge to agricultural productivity in regions where saline soils are prevalent (Ehtaiwesh et al. 2024).

The mechanisms underlying salt tolerance in wheat have been extensively investigated (Jiang et al. 2021; Tibesigwa et al. 2025). However, most previous studies have focused on the effects of NaCl stress (Saddiq et al. 2021). This has provided valuable insights into the responses of plants to one of the most common forms of soil salinity (Tibesigwa et al. 2025). However, a comprehensive understanding of wheat adaptation to a broader range of saline conditions necessitates the examination of other salts, such as alkaline salt and Na_2_SO_4_, that also contribute to soil salinity and have distinct effects on plant physiology (Kamran et al. 2020; Aniskina et al. 2023).

In this study, we explored the effects of NaCl, Na_2_SO_4_, NaHCO_3_, and Na_2_CO_3_ on the growth and physiology of wheat seedlings. The plants were exposed to equivalent Na^+^ concentrations under four treatments: NaCl, Na_2_SO_4_, NaHCO_3_, and Na_2_CO_3_. We aimed to uncover the regulatory mechanisms of wheat exposed to various salinities, including antioxidation, osmotic regulation, and ion balance regulation mechanisms. Our findings provide an important foundation for understanding the distinct mechanisms underlying salt and alkaline stress in wheat, which may aid future breeding efforts to improve wheat salt tolerance.

## 2 Materials and Methods

### 2.1 Plant Culture and Experimental Design

First, wheat seeds (Ji Mai 22) were sterilized with 75% alcohol for 10 min and then washed three times with distilled water (Pan et al. 2020; Wang, Wang, et al. 2020). The sterilized seeds were germinated in a plastic nursery box in an artificial climate chamber. Five‐day‐old seedlings were transferred to a black plastic incubator containing 1 L of 1/2 Hoagland nutrient solution. The solutions were constantly inflated using an air pump and replaced every 2 days; the pH of the solutions was adjusted using NaOH and HCl. The seeds were illuminated using LED and maintained at 60% humidity and a fixed temperature of 22°C during the day and 18°C during the night. After 20 days of acclimatization under control conditions, five experimental treatments were performed: CK (no NaCl, Na_2_SO_4_, Na_2_CO_3_, and NaHCO_3_), 60 mM Na_2_SO_4_, 120 mM NaCl, 60 mM Na_2_CO_3_, and 120 mM NaHCO_3_.

### 2.2 Determination of Plant Growth

After the experimental treatments, quantitative documentation of the selected growth parameters was systematically performed (Nassar et al. 2020). Specifically, the roots and shoots of wheat were carefully separated, and their fresh weights were determined using a digital scale with a precision of 0.01 g (g). Subsequently, the roots and shoots of wheat were dried at 85°C for 48 h in an oven. In addition, the length of the roots and the overall height of various wheat varieties were precisely measured using a millimeter ruler.

### 2.3 Determination of Membrane Injury

Membrane injury (MI) was assessed by measuring the electrolyte leakage (Jiang et al. 2020). Shoots and root sections were obtained from randomly selected seedlings from each treatment group. The sections were meticulously washed with deionized water to remove any surface‐adhered electrolytes. Subsequently, approximately 1 g of fresh sample was immersed in 15 mL glass test tubes containing 10 mL of deionized water. The samples were maintained at a temperature of 25°C for 2 h, and the electrical conductivity (R1) was determined. Subsequently, the samples were boiled for 1 h and cooled to room temperature, and the electrical conductivity was recorded again (R2). The MI index was calculated using the following formula: MI (%) = R1/R2 × 100% (Wei et al. 2015).

### 2.4 Determination of Antioxidant Enzyme Activities

Wheat tissues were homogenized in liquid nitrogen. Subsequently, a precise aliquot (0.1 g) from the ground tissue sample was weighed and transferred to a 15 mL centrifuge tube containing 10 mL of phosphate‐buffered saline (PBS). The mixture was thoroughly vortexed and subsequently centrifuged at 12,000 *g* for 20 min at 4°C. The resulting supernatant was collected to determine the activities of various antioxidant enzymes.

Superoxide dismutase (SOD) activity was determined using the nitroblue tetrazolium (NBT) assay (Pan, Ding, et al. 2022). Briefly, 0.1 mL of the enzyme extract was mixed with 1.5 mL of PBS, 0.3 mL of NBT solution (750 μM), 0.3 mL of riboflavin solution (20 μM), 0.3 mL of EDTA‐2Na solution (100 μM), and 0.3 mL of L‐methionine (L‐Met) solution (130 μM). SOD activity was determined based on the reduction of NBT, by measuring the absorbance at 560 nm (Kamran et al. 2021). Peroxidase (POD) activity was assessed using the guaiacol assay. Specifically, 0.1 mL of enzyme extract was combined with 0.9 mL of PBS, 1 mL of hydrogen peroxide (12% v/v), and 1 mL of guaiacol (50 mM). The absorbance was measured at 470 nm to assess the catalytic activity of the enzyme.

The activity of ascorbate peroxidase (APX) was determined by monitoring the change in absorbance at 290 nm, which corresponds to the oxidation of ascorbate (Wang, Wang, et al. 2020). Briefly, 0.1 mL of the enzyme extract was added to an APX reaction mixture containing PBS, 0.1 mL of H_2_O_2_ (20 mM, 0.1 mL), and ascorbate (5 mM), and the absorbance was determined using a spectrophotometer. Catalase (CAT) activity was determined by measuring the changes in absorbance at 240 nm. Specifically, 0.1 mL of the enzyme extract was added to a 3 mL CAT reaction mixture system with 1.9 mL PBS and 1 mL H_2_O_2_ (100 mM), and absorbance was determined using a spectrophotometer.

### 2.5 Determination of Lipid Peroxidation

Fresh samples of wheat (0.1 g) were selected and homogenized in trichloroacetic acid (TCA) supplemented with 0.50% 2‐thiobarbituric acid (TBA). After incubating at 100°C for 20 min, the solution was immediately cooled to 25°C, and then centrifuged at 5000 *g* for 10 min. The absorbance of the mixture was measured at 600, 532, and 450 nm.

### 2.6 Determination of Osmotic Adjustment Substances

Approximately 0.1 g of fresh samples of wheat were selected to measure the proline content of tissues. The samples were placed in tubes containing 10 mL of 3% aqueous sulfosalicylic acid and then incubated for 10 min. A reaction mixture containing 2 mL of 6 mM orthophosphoric acid, 2 mL of acidic ninhydrin, and 2 mL of glacial acetic acid was prepared. Then 2 mL of the filtrate was added to the mixture, which was boiled at 100°C and then cooled to room temperature. Next, toluene (4 mL) was added to the solution and vortexed for 10 min. The absorbance was measured at 520 nm, and the proline content was calculated using a correlation formula.

The total soluble sugar content of fresh wheat was determined using the anthrone‐sulfuric acid method (Su et al. 2020). To extract carbohydrates from the fresh sample, 0.1 g fresh plant tissue was transferred to glass tubes containing 10 mL of distilled water and boiled for 30 min. The extract was mixed with 0.5 mL of anthrone, 1.5 mL of distilled water, and 5 mL of concentrated sulfuric acid. After boiling for 1 min, the absorbance of each sample was measured at 625 nm.

The soluble protein levels in the diverse plants were determined according to a previous method (Wang, Gao, et al. 2020). Briefly, 0.1 g of the fresh samples was added to 5 mL of PBS and homogenized. Subsequently, 0.1 mL of the homogenate was combined with 0.9 mL of distilled water and 5 mL G250. The absorbance was measured at 595 nm. The betaine content of the wheat was assayed using a previously described method. Fresh samples (0.1 g) were homogenized in 2 mL of distilled water and incubated in a shaking incubator for 24 h at 150 *g*. After centrifuging at 10,000 *g* for 20 min, the pH of the supernatant was adjusted using hydrochloric acid. Subsequently, 0.5 mL of the supernatant was added to 0.5 mL of 3% saturated Lehman's salt solution at 4°C for 5 h. After centrifuging at 10,000 *g* for 15 min, the supernatant was discarded, the precipitate was washed three times with ether, and then solubilized in 2 mL of 70% acetone. The absorbance was measured at 525 nm.

### 2.7 Determination of H_2_O_2_ and $O_{2}^{\cdot -}$

The H_2_O_2_ concentrations in various wheat tissues were determined according to a previous study (Pan, Ding, et al. 2022). The experiment was based on measuring the change in the absorbance of the titanium peroxide complex at 415 nm. The absorbance values were quantified using a standard curve generated using known concentrations of H_2_O_2_. The hydroxylamine oxidation method was used to determine the contents of $O_{2}^{\cdot -}$ of tissues (Pan, Buitrago, et al. 2022). Briefly, 0.5 mL of antioxidant enzyme extract (Section 2.6) was mixed with 1 mL of hydroxylamine and incubated at 100°C for 1 h. Thereafter, 1 mL of 7 mM α‐naphthylamine and 17 mM P‐aminobenzene sulfonic solution was added to the mixture and incubated for 20 min. Absorbance was recorded at 530 nm, and the $O_{2}^{\cdot -}$ content in tissues was quantified using a linear calibration curve of NaNO_2_.

### 2.8 Determination of Contents of K, Na, Ca, and Mg

The contents of the inorganic elements (K, Na, Ca, and Mg) were determined using approximately 0.25 g of dried sample (Zheng et al. 2025). Wheat tissues were digested using electric heating equipment using concentrated nitric acid (HNO_3_) and then cooled to room temperature. Subsequently, each clear digest was quantitatively introduced into 50 mL volumetric flasks. The elemental contents were determined using inductively coupled plasma mass spectrometry (ICP‐MS).

### 2.9 Expression Profiling of the Diverse Gene

The qPCR was performed according to our previous study (Tong et al. 2024), and the primer was displayed at Table S1. The expression of the *TaSOD* gene family was determined according to a previous study (Jiang et al. 2024). Briefly, RNA‐Seq data under salt stress were retrieved from WheatOmics 1.0 (http://wheatomics.sdau.edu.cn/). *TaSOD* expression data across various wheat tissues were obtained from the Wheat Expression Browser (https://www.wheat‐expression.com/). The number of *SOD* genes in different plants was determined from the Public PLAZA database (https://bioinformatics.psb.ugent.be/plaza/) (Van Bel et al. 2022).

### 2.10 Statistical Analyses

The experiment followed a randomized complete block design with four biological replicates. All data were analyzed using a one‐way analysis of variance (ANOVA) using SPSS 27.0 (SPSS Inc., Chicago, USA). The treatment mean values were determined using the least significant difference (LSD) test, and differences were considered significant at *p* < 0.05.

## 3 Results

### 3.1 Alkaline Salts Cause More Severe Growth Inhibition Than Neutral Salts

**FIGURE 1:**
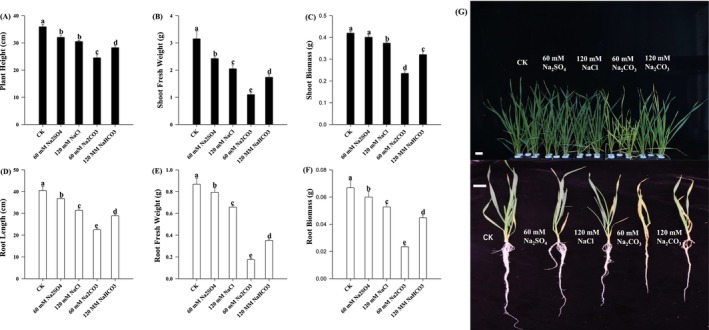
Plant height (A), shoot fresh weight (B), shoot biomass (C), root length (D), root fresh weight (E), root biomass (F), and phenotype image (G) of wheat seedlings. Bar = 5 cm. Different letters indicate significant differences (*p* < 0.05).

After the treatment, the growth status of the plants was investigated. Salt and alkaline treatments significantly reduced wheat seedling growth (Figure 1). Wheat seedlings exhibited significantly reduced height, root length, fresh weight, and biomass under salt stress conditions. Compared to neutral salt stress, alkaline stress showed more pronounced inhibitory effects on all measured parameters. These parameters reached their lowest values under the 60 mM Na_2_CO_3_ treatment.

### 3.2 Chlorophyll Degradation Is Exacerbated Under Alkaline Stress

**FIGURE 2:**
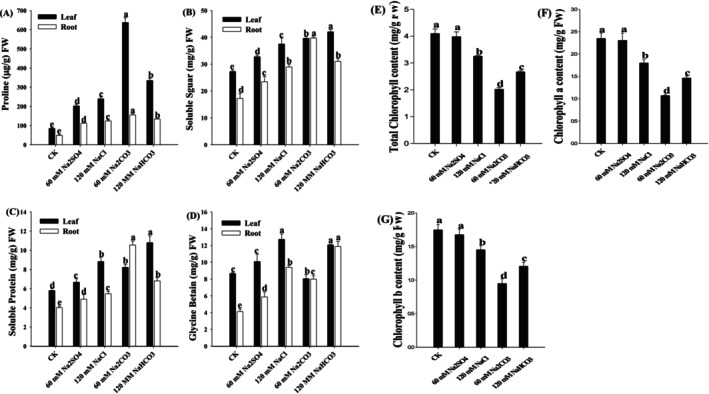
Proline (A), soluble sugar (B), soluble protein (C), and glycine betaine (D) in the leaves and roots of wheat seedlings. Total chlorophyll content (E), chlorophyll a content (F), and chlorophyll b content (G) of leaves of wheat seedlings. Different letters indicate significant differences (*p* < 0.05).

Salt and alkaline stress induced chlorophyll degradation in wheat seedling leaves, thereby reducing the chlorophyll content in plants (Figure 2). At equivalent stress concentrations, alkaline stress caused a more pronounced decline in chlorophyll a, chlorophyll b, and total chlorophyll than salt stress. The lowest levels were found under 60 mM Na_2_CO_3_ treatment, with the chlorophyll a, chlorophyll b, and total chlorophyll decreasing to 50%, 49%, and 48% (*p* < 0.01) compared to the control, respectively.

### 3.3 Osmolyte Accumulation Reveals Stress‐Specific and Organ‐Specific Metabolic Reprogramming

Salt and alkaline stress significantly upregulated the biosynthesis of osmoregulatory substances in wheat seedlings, with alkaline stress inducing a markedly greater accumulation than salt at equivalent concentrations (Figure 2). Proline exhibited extreme alkaline sensitivity, reaching 7.5‐fold (roots) and 3.2‐fold (leaves) higher levels than that of the controls under 60 mM Na_2_CO_3_ (*p* < 0.01). Soluble sugar partitioning also displayed organ‐specific optimization: root sugars reached their highest levels (2.31× control, *p* < 0.01) under 60 mM Na_2_CO_3_, whereas foliar sugars peaked at 1.54× control under120 120 mM NaHCO_3_ (*p* < 0.01). Similarly, alkaline salts potentiated soluble protein synthesis, with foliar proteins reaching 1.54× control levels under 120 mM NaHCO_3_ (*p* < 0.01). Notably, betaine regulation differed between organs: 120 mM NaCl increased foliar betaine to 1.47× the controls (*p* < 0.01), while root betaine surged to 2.88× the controls (*p* < 0.01) under 120 mM NaHCO_3_, indicating tissue‐specific metabolic reprogramming under ionic heterogeneity.

### 3.4 The Antioxidant Enzymatic Response Is Tailored to the Specific Ion

**FIGURE 3:**
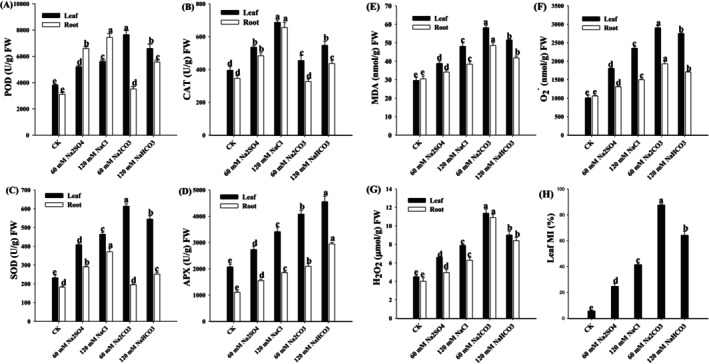
POD (A), CAT (B), SOD (C), and APX (D) in the leaves and roots of wheat seedlings. MDA (E), $O_{2}^{\cdot -}$ (F), H_2_O_2_ (G), and leaf MI (H) in the leaves and roots of wheat seedlings. Different letters indicate significant differences (p <0.05).

Salt and alkaline stress induced variations in antioxidant enzyme activity in wheat seedling leaves and roots (Figure 3). The peroxidase (POD) activity in leaves reached its maximum value at 2.01 times that of the control (*p* < 0.01) under 60 mM Na_2_CO_3_ stress, whereas root POD activity peaked at 1.79 times the control (*p* < 0.01) under 120 mM NaCl stress. Under equivalent stress concentrations, the catalase (CAT) activity in the leaves and roots of plants exposed to alkaline stress was lower than that in plants exposed to salt stress. The maximum CAT activity in the leaves and roots under 120 mM NaCl stress was 1.37‐ and 1.65‐fold higher than that of the control (*p* < 0.01), respectively. Furthermore, compared to the control, SOD activity in the leaves peaked at 2.64 times under 60 mM Na_2_CO_3_ stress and 2.03 times under 120 mM NaCl stress. Notably, APX activity in leaves and roots under alkaline stress exceeded that under salt stress at equivalent concentrations. The highest APX activity was observed under 120 mM NaHCO_3_ stress, reaching 2.2‐ and 2.66‐fold increases compared with the control in the leaves and roots, respectively (*p* < 0.01).

### 3.5 Alkaline Stress Induces More Severe Oxidative Damage and Membrane Injury

Salt and alkaline stresses induced significant increases in MDA, $O_{2}^{\cdot -}$, and H_2_O_2_ contents in wheat seedling leaves (Figure 3). Under equivalent stress concentrations, alkaline stress resulted in a higher accumulation of these oxidative stress markers than salt stress. The maximum values were observed under 60 mM Na_2_CO_3_ treatment, reaching 1.97‐fold (MDA), 2.88‐fold ($O_{2}^{\cdot -}$), and 2.53‐fold (H_2_O_2_) that of the control levels (*p* < 0.01). Similarly, the root system exhibited analogous responses, with alkaline salt stress inducing a greater increase in the levels of MDA, $O_{2}^{\cdot -}$, and H_2_O_2_ relative to neutral salt stress. The 60 mM Na_2_CO_3_ treatment induced peak values corresponding to 1.59‐fold (MDA), 1.82‐fold ($O_{2}^{\cdot -}$), and 2.72‐fold (H_2_O_2_) increases compared to the control (*p* < 0.01). Furthermore, salt and alkaline stress significantly enhanced plasma membrane permeability in wheat seedling leaves, with alkaline stress demonstrating more pronounced effects than salt stress at equivalent concentrations. The maximum membrane permeability was recorded under 60 mM Na_2_CO_3_ stress, showing a 15.5‐fold increase relative to the control (*p* < 0.01).

### 3.6 Ion Homeostasis Is Differentially Disrupted, Defining Distinct Toxicity Profiles for Neutral and Alkaline Salts

**FIGURE 4:**
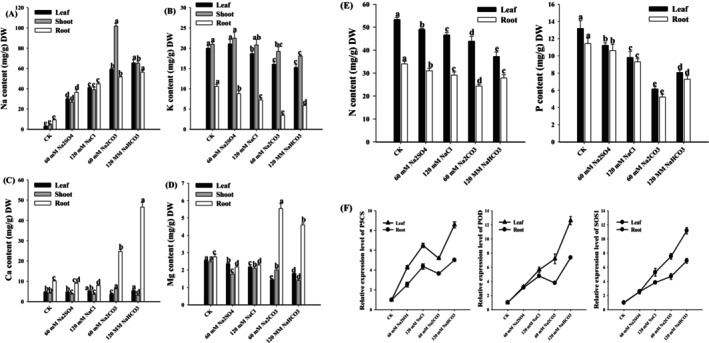
Na (A), K (B), Ca (C), and Mg (D) in the leaves, shoots, and roots of wheat seedlings. Element content (E) and gene expression (F) content in the leaves and roots of wheat seedlings. Different letters indicate significant differences (*p* < 0.05).

Salt and alkaline stress induce substantial sodium (Na) accumulation in the leaves, stems, and roots of wheat seedlings (Figure 4). Notably, alkaline stress resulted in significantly higher Na accumulation across all examined tissues than neutral salt stress at equivalent concentrations. Under 60 mM Na_2_CO_3_ stress, the stem exhibited the most pronounced Na accumulation, reaching 23.3‐fold that of the control level (*p* < 0.01). Maximum Na accumulation in leaves (21.4‐fold) and roots (6.08‐fold) relative to controls was observed under 120 mM NaHCO_3_ stress (*p* < 0.01 for both). Concurrently, the potassium (K) content of wheat seedlings was significantly depleted under both stress conditions, with alkaline stress inducing a more severe K reduction than salt stress at comparable concentrations. The minimum K levels in leaves (76.2% of control) and stems (86.2% of control) were recorded under 120 mM NaHCO_3_ stress (*p* < 0.01), while root K content reached its lowest value (32.3% of control) under 60 mM Na_2_CO_3_ stress (*p* < 0.01). Alkaline stress triggered significant calcium (Ca) enrichment in plant tissues. The most substantial Ca accumulation occurred in roots under 120 mM NaHCO_3_ stress (4.59‐fold of control, *p* < 0.01), followed by stems under 60 mM Na_2_CO_3_ stress (1.51‐fold, *p* < 0.01), with leaves showing moderate Ca elevation (1.13‐fold, *p* < 0.05) under 120 mM NaHCO_3_ conditions. Magnesium (Mg) distribution exhibited tissue‐specific responses. While alkaline stress enhanced root Mg content, peaking at 3.14‐fold that of control under 60 mM Na_2_CO_3_ (*p* < 0.01), it significantly reduced Mg levels in aerial tissues. Minimum Mg concentrations were observed in leaves (56.4% of control) under 60 mM Na_2_CO_3_ stress and stems (56.7% of control) under 120 mM NaHCO_3_ stress (*p* < 0.01 for both). These findings collectively demonstrate that differential ion homeostasis may respond to saline‐alkaline stress conditions in wheat seedlings.

### 3.7 Macronutrient Depletion Is a Hallmark of Alkaline Stress Toxicity

Salt and alkali stress reduced nitrogen (N) content in the leaves and roots of wheat plants (Figure 4). Under equivalent stress concentrations, alkaline stress caused a more pronounced N depletion than salt stress. The lowest N content in leaves was observed under 120 mM NaHCO_3_ treatment, corresponding to 69.7% of the control (*p* < 0.01), whereas roots exhibited the lowest N content (49.6% of the control) under 60 mM Na_2_CO_3_ stress (*p* < 0.01). Similarly, the phosphorus (P) content in both tissues decreased under salt and alkali stress, with alkaline stress inducing greater reductions than salt stress at equivalent concentrations. The most severe P depletion occurred under 60 mM Na_2_CO_3_ treatment, with leaf and root P content reaching minimum values of 46.6% and 45.7% of the control, respectively (*p* < 0.01).

### 3.8 Expression Dynamics of *TaSOD* and Ion Homeostasis Genes Underscore Genotype‐ and Stress‐Specific Adaptation

**FIGURE 5:**
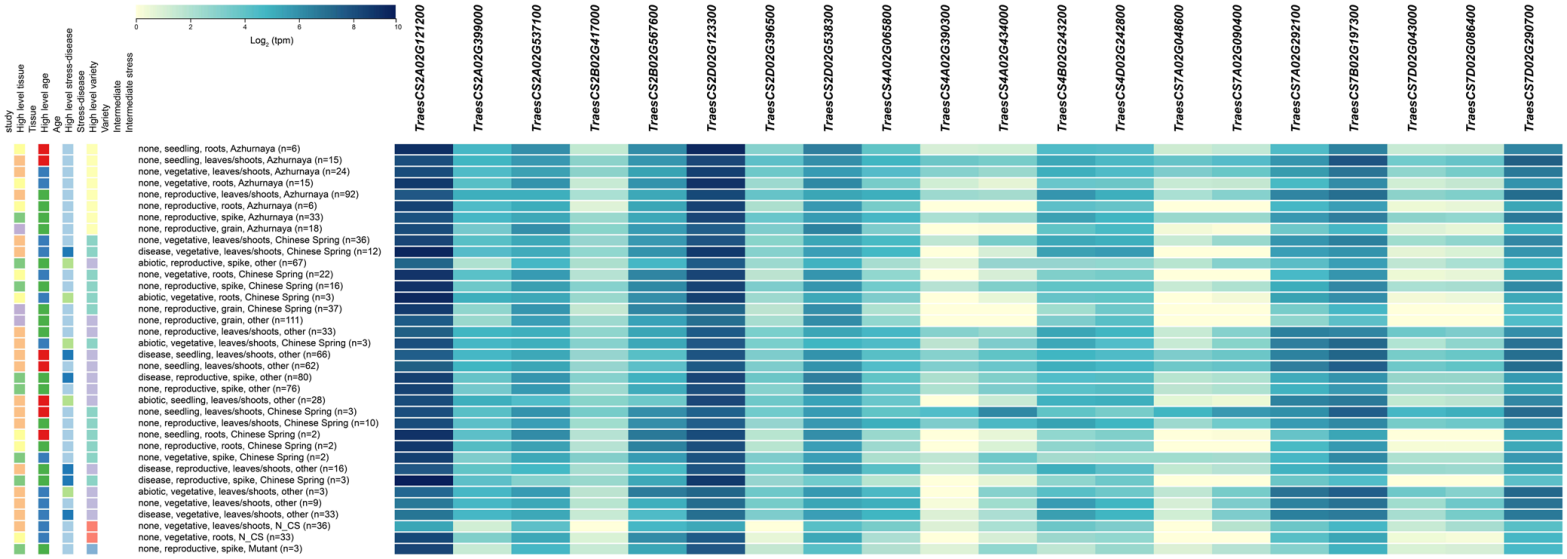
Expression of *TaSOD* genes in various wheat tissues.

**FIGURE 6:**
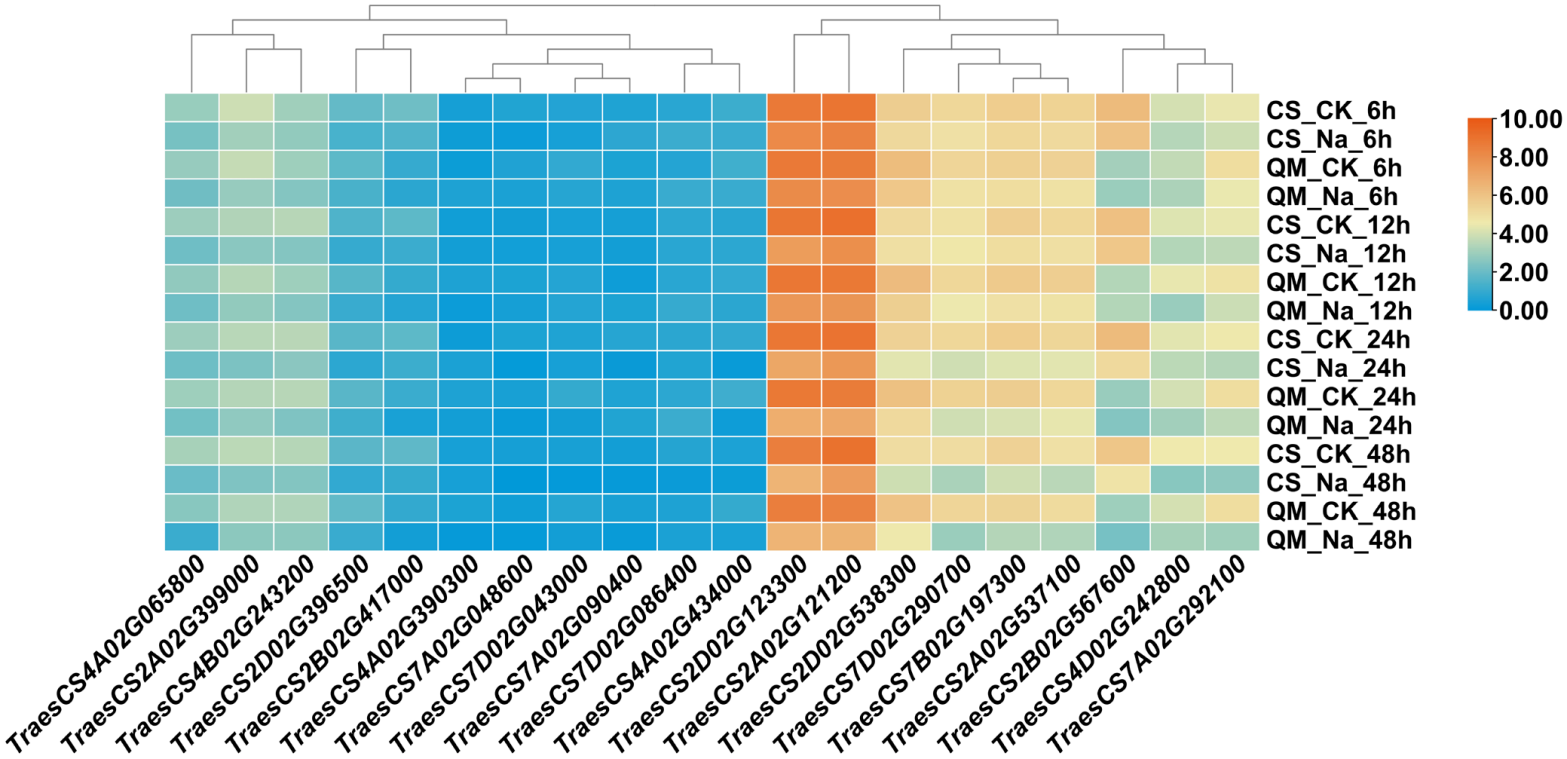
Expression of *TaSOD* genes in Chinese Spring (CS) and Qing Mai 6 (QM) under salt stress.

The expression of *P5CS*, *POD*, and *SOS1* in leaves and roots was evaluated under diverse saline‐alkali treatments. Compared with the control, the expression of *SOS1* genes was induced under saline‐alkali stress. Notably, 20 genes displayed distinct expression patterns in response to diverse developmental conditions (Figure 5). Interestingly, *TraesCS2A02G121200* and *TraesCS2D02G123300* showed high expression, and *TraesCS4A02G390300*, *TraesCS7A02G048600*, and *TraesCS7D02G043000* demonstrated low transcript levels in different tissues, indicating their multiple functions in wheat growth and development. Half of the *TaSOD* genes were highly expressed in response to salt stress in the Chinese Spring (CS) and Qing Mai 6 (QM) wheat (Figure 6). Furthermore, under the control and salt treatments, *TraesCS2B02G567600* expression was lower in QM wheat than in CS wheat. All the transcripts of *TaSOD* were decreased after salt treatment in both CS and QM wheat. Over time, the expression of these genes gradually decreased. Therefore, *TaSOD* may be a key gene responsible for the difference in the response to salt stress between the two wheat genotypes.

**FIGURE 7:**
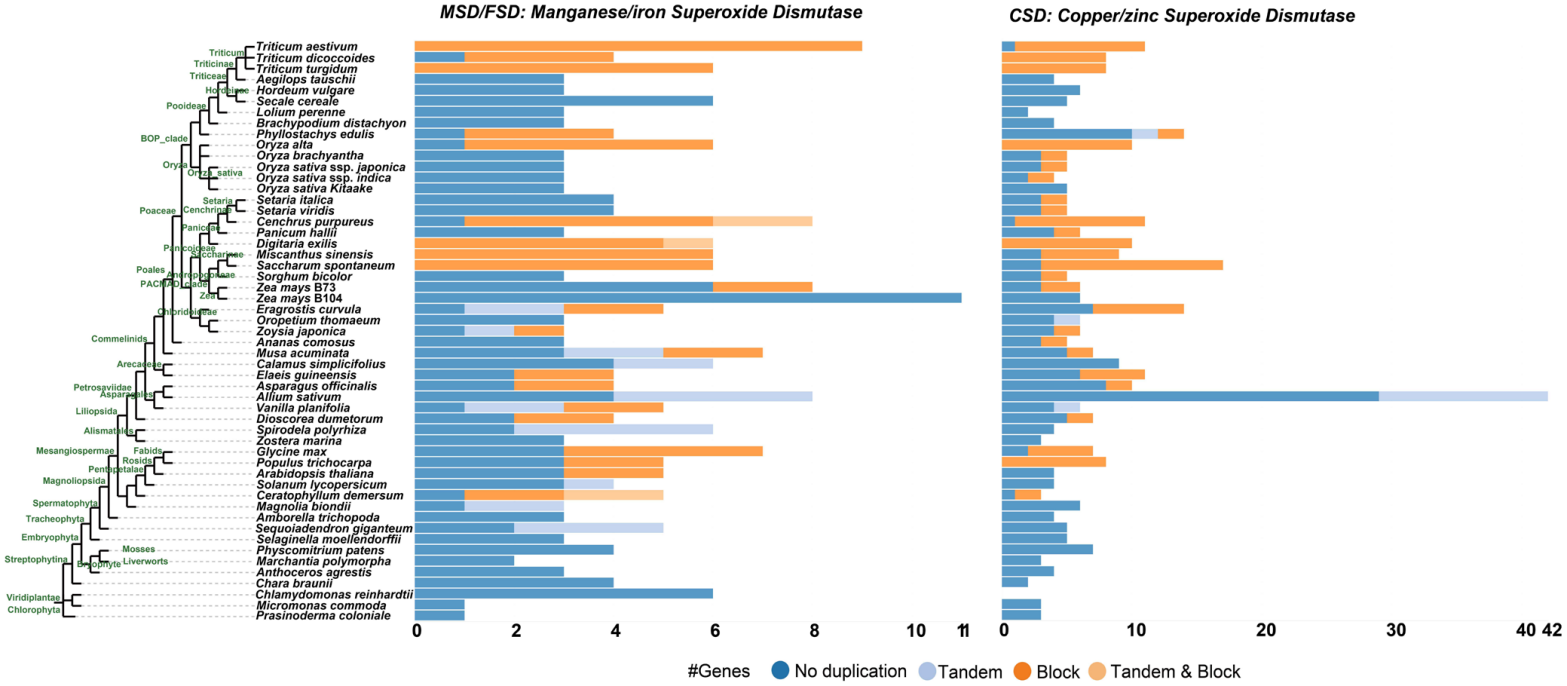
Evolutionary analysis of the *SOD* gene family across plants. Tandem and block gene duplicates of the *SOD* gene family in *Chlorophyta* and *Embryophyta*.

Moreover, we analyzed the evolution of the plant *SOD* gene family using public databases (Figure 7) (https://bioinformatics.psb.ugent.be/plaza.dev/instances/monocots_05/genes/gene_duplication_analysis/interpro/IPR036324, https://bioinformatics.psb.ugent.be/plaza.dev/instances/monocots_05/genes/gene_duplication_analysis/interpro/IPR001424). A total of 240 and 369 *MSD/FSD* and *CSD* genes, respectively, were confirmed in 53 green plants, including *Chlorophyta* and *Embryophyta*, using the PLAZA database. The percentages of tandem and block gene duplicates in the *MSD/FSD* gene family were 10% and 31%, respectively. Furthermore, the number of tandem and block gene duplicates in the *CSD* gene family was 19 and 132, respectively. The *CSD* gene family expanded in monocots (e.g., *T. aestivum* and *Cenchrus purpureus* ), eudicots (e.g., *Glycine max* and *Populus trichocarpa* ), bryophytes, and ferns, but not in algae, indicating that the expansion of the *CSD* gene family may date back to the emergence of land plants. However, *MSD/FSD* genes have not been duplicated in many plants, including *Sorghum bicolor* and *Hordeum vulgare* . Thus, *SOD* is highly conserved in green plants and may expand in terrestrial plants.

## 4 Discussion

### 4.1 Differential Growth Inhibition and Ion‐Specific Toxicity Underlie Distinct Salt Stress Profiles

NaCl, Na_2_SO_4_, NaHCO_3_, and Na_2_CO_3_ stress significantly reduced the growth of wheat seedlings, as indicated by the decreased length of shoot and root, as well as a reduction in the fresh and dry biomass (Zhang et al. 2020). However, NaHCO_3_ and Na_2_CO_3_ stress led to a more pronounced inhibition of shoot elongation, while Na_2_SO_4_ had a slightly greater impact on root development (Figure 1). This differential inhibition may be attributed to the varying uptake and distribution of Na^+^ and ${\mathrm{SO}}_{4}^{2-}$ ions, and their effects on cellular osmosis and nutrient transport systems (Saddiq et al. 2021). Membrane injury, quantified by electrolyte leakage, further confirms the severity of the stress imposed by diverse salt types (Fang et al. 2021). Interestingly, NaHCO_3_‐ and Na_2_CO_3_‐ treated seedlings showed higher levels of membrane injury than those under NaCl and Na_2_SO_4_ stress (Figure 3), suggesting greater disruption of cellular membranes. This could be due to the high osmotic potential and toxic effects of ${\mathrm{HCO}}_{3}^{-}$ and ${\mathrm{CO}}_{3}^{2-}$ ions, which interfere with ion transporters and disrupt membrane integrity. In contrast, ${\mathrm{SO}}_{4}^{2-}$ ions, although deleterious, might be better tolerated due to their involvement in essential metabolic processes, including sulfur assimilation and amino acid biosynthesis. Furthermore, we used equimolar Na^+^ concentrations in a hydroponic system, which were designed to isolate the specific ionic effects of Cl^−^, ${\mathrm{SO}}_{4}^{2-}$, ${\mathrm{HCO}}_{3}^{-}$, and ${\mathrm{CO}}_{3}^{2-}$ under controlled conditions (Wang, Shen, et al. 2022). The chosen concentration represents a physiologically significant stress level that reliably triggers key adaptive and injury responses in wheat seedlings, facilitating a comparative mechanistic analysis. Although this approach may not fully replicate the long‐term, composite stress dynamics of saline‐alkali fields (Song, Liu, et al. 2025), it provides a fundamental and necessary understanding of how different salt anions drive distinct physiological pathways.

### 4.2 Antioxidant Defense Systems Are Tailored to the Specific Oxidative Challenge of Each Salt Type

Alkaline stress has been extensively studied in several crops, including rice (Ganapati et al. 2022), tomato (Xu et al. 2022), soybean (He et al. 2025), and cotton (Fan et al. 2021). However, the physiological and molecular mechanisms underlying its action in wheat remain unclear. The generation of ROS, such as H_2_O_2_ and $O_{2}^{\cdot -}$, is a hallmark of alkaline and salt stress. The accumulation of ROS induces oxidative damage to proteins and lipids, necessitating an efficient antioxidant defense system (Yu et al. 2024). In our study, all types of stress triggered elevated activities of important antioxidant enzymes, such as SOD, CAT, POD, and APX (Figure 3). The activity of SOD, the primary scavenger of $O_{2}^{\cdot -}$, was notably higher in NaCl‐treated seedlings compared to Na_2_SO_4_, reflecting a more intense oxidative burst induced by Cl^−^ stress. Similarly, CAT and APX activities were upregulated in distinct treatments, with NaCl eliciting a stronger response. This enzymatic upregulation suggests that wheat seedlings attempt to mitigate ROS accumulation through a coordinated antioxidant mechanism, particularly under severe oxidative conditions associated with NaCl stress. Interestingly, POD activity was markedly higher in Na_2_SO_4_‐treated plants, implying that POD might play a vital critical role in mitigating ${\mathrm{SO}}_{4}^{2-}$‐related stress. Besides, compared to a stronger CAT induction under NaCl stress, the POD activity elevated under Na_2_SO_4_ stress alongside a relatively lower CAT response (Figure 3), which might reflect the activation of distinct ROS scavenging pathways to the oxidative damage in wheat (Ren and Chen 2025). Therefore, it is essential to explore the concepts of enzyme substrate specificity, subcellular localization, and potential compensatory mechanisms in the antioxidant network (Song, Li, et al. 2025; Wang, Shen, et al. 2022). These results imply that wheat activates salt‐specific antioxidant pathways, tailoring its response to the nature of the ionic stressor (Wang, Gao, et al. 2020).

### 4.3 The Evolution and Stress‐Responsive Expression of SOD Gene Family Suggest Its Role in Salinity Adaptation

*TaSOD* plays an essential role in the development and diverse stresses including salt (Wang et al. 2021), drought (Saed‐Moucheshi et al. 2021), and heat (Kumar et al. 2020). For instance, *TaSOD2* overexpression enhances SOD activity in both wheat and *Arabidopsis thaliana* , which displays Cu/Zn‐SOD enzymatic activity in vitro, confirming that *TaSOD2* functions as a Cu/Zn‐SOD (Wang et al. 2016). Besides, ectopic expression of *TaSOD5* improves salt stress tolerance in *A. thaliana* (Wang et al. 2021). Increasing SOD activity through gene overexpression improves tolerance to abiotic stress by inducing ROS‐scavenging capacity (Gill et al. 2015). Furthermore, transgenic lines increase the resistance to H_2_O_2_ and reduce intracellular H_2_O_2_ levels. Therefore, *TaSOD* may enhance salt stress tolerance by strengthening the oxidative stress defense system of plants. Taken together, our findings indicate an essential role for *TaSOD* in the response to salt and alkaline stress (Wang, Wang, et al. 2020). The differential expression patterns of *TaSOD* genes observed in public transcriptomes correlate with our physiological measurements of oxidative stress (Figure 7), suggesting their potential involvement. Previous work has employed qPCR to confirm the response to salt stress (Jiang et al. 2019), which is similar to the results of the public database. However, further studies are required to confirm the functions of these candidate genes through gene editing (Qin et al. 2025). Furthermore, integrating multi‐omics data with physiological traits will further elucidate crop responses to alkaline stress (van Zelm et al. 2020).

## 5 Conclusion

In conclusion, this study provides a comprehensive comparative analysis of the physiological and molecular responses of wheat seedlings to distinct types of salt stress (NaCl, Na_2_SO_4_, NaHCO_3_, and Na_2_CO_3_). Besides, NaCl and Na_2_SO_4_ primarily induced ionic toxicity and osmotic stress, triggering a strong upregulation of ion transporter genes (e.g., *SOS1*). In contrast, NaHCO_3_ and Na_2_CO_3_ imposed combined ionic, high‐pH (alkaline), and oxidative stress, which activated a more complex defense network involving enhanced antioxidant enzyme activities (e.g., CAT, POD) and specific pH‐regulation mechanisms. Our principal findings demonstrate that antioxidant defenses are anion‐tailored and ion homeostasis is differentially remodeled. The mechanistic insights and candidate genes identified herein establish a vital foundation for breeding wheat varieties with enhanced and broad‐spectrum tolerance to complex saline‐alkaline environments.

## Author Contributions

**Duo Liu:** formal analysis, writing original draft. **Zheng Wang:** investigation, formal analysis, writing original draft. **Hongyao Lou:** formal analysis. **Ping Li:** formal analysis. **Kangfeng Cai:** review and editing. **Wei Jiang:** review and editing. **Zhaobo Chen:** conceptualization, formal analysis, writing review and editing, project administration, resources, supervision.

## Ethics Statement

The authors have nothing to report.

## Consent

Written informed consent was obtained from all study participants.

## Conflicts of Interest

The authors declare no conflicts of interest.

## Supporting information


**Table S1:** The primer used in this study.

## Data Availability

Research data are not shared.
